# A cross-sectional survey of Department of Veterans Affairs laboratory practices for identification of carbapenem-resistant *Acinetobacter baumannii* and *Pseudomonas aeruginosa*

**DOI:** 10.1017/ash.2024.404

**Published:** 2024-11-11

**Authors:** Aubrey M. Sawyer, Cara Ray, J. Stacey Klutts, Margaret Fitzpatrick, Katie J. Suda, Natalie Hicks, Martin Evans, Makoto Jones, Christopher D. Pfeiffer, Charlesnika T. Evans, QUERI CARRIAGE Program

**Affiliations:** 1 Department of Veterans Affairs, Center of Innovation for Complex Chronic Healthcare, Edward Hines, Jr. VA Hospital, Hines, IL, USA; 2 Department of Medicine, Division of Infectious Diseases, Northwestern University Feinberg School of Medicine, Chicago, IL, USA; 3 Department of Pathology, University of Iowa Carver College of Medicine, Iowa City, IA, USA; 4 Department of Veterans Affairs, Iowa City VA Health Care System, Iowa City, IA, USA; 5 Center of Innovation for Veteran Centered and Value Driven Care, Rocky Mountain Regional VA Medical Center, Aurora, CO, USA; 6 University of Colorado Anschutz Medical Campus, Aurora, CO, USA; 7 Center for Health Equity Research and Promotion, Pittsburgh VA Medical Center, Pittsburgh, PA, USA; 8 School of Medicine, University of Pittsburgh, Pittsburgh, PA, USA; 9 MDRO Prevention Office, VA National Infectious Diseases Service, Washington, DC, USA; 10 Department of Veterans Affairs, VA Salt Lake City Healthcare System, Salt Lake City, UT, USA; 11 Department of Medicine, Division of Epidemiology, University of Utah, Salt Lake City, UT, USA; 12 Department of Veterans Affairs, Portland VA Healthcare System, Portland, OR, USA; 13 Department of Medicine, Division of Infectious Diseases, Oregon Health Science University, Portland, OR, USA; 14 Center for Health Services and Outcomes Research and Department of Preventive Medicine Institute for Public Health and Medicine, Northwestern University Feinberg School of Medicine, Chicago, IL, USA

## Abstract

Control of carbapenem-resistant *Acinetobacter baumannii* and *Pseudomonas aeruginosa* spread in healthcare settings begins with timely and accurate laboratory testing practices. Survey results show most Veterans Affairs facilities are performing recommended tests to identify these organisms. Most facilities report sufficient resources to perform testing, though medium-complexity facilities report some perceived barriers.

## Introduction

Carbapenem-resistant *Acinetobacter baumannii* (CRAB) and *Pseudomonas aeruginosa* (CRPA) are rapidly emerging multidrug-resistant organisms (MDRO) with high morbidity and mortality and increasingly limited treatment options.^1^ In fact, these organisms have been listed as “critical” threats by the Centers for Disease Control and Prevention and the World Health Organization.^2,3^ Infections by CRAB and CRPA are frequently seen in hospitals, causing thousands of deaths and millions of dollars in excess healthcare spending annually.^4,5^

Timely and accurate identification is necessary for reducing the transmission of MDROs through the implementation of infection control measures. Knowledge of laboratory practices for testing and identification is essential to optimize that approach. The Veterans Health Administration (VHA) released guidelines for Veterans Affairs (VA) medical centers (VAMCs) for other carbapenem-resistant organisms.^6,7^ However, it is unknown if laboratories have consistent testing practices for CRAB and CRPA. This study reports the results of a survey of VAMC laboratories to assess the reported incidence of CRAB and CRPA, testing and identification practices used, and availability of resources for testing at each facility.

## Methods

An electronic cross-sectional survey was distributed to VA laboratory staff email groups representing up to 126 VA facilities in September–October 2023. Survey questions examined CRAB and CRPA incidence, testing practices, and available resources (Supplement). The survey was developed in collaboration with the VA MDRO Program Office and administered through VA Research Electronic Data Capture (REDCap).^8^

Survey responses were compared by facility location (urban vs rural) and patient complexity as classified by the VHA facility complexity model (high vs medium vs low) using Fisher’s exact tests. Complexities 1a–1c were classified as high-complexity and represent facilities with high-level intensive care unit (ICU) patients, while level 2 and 3 facilities were classified as medium-complexity and low-complexity, respectively, and represent lower-volume, less complex patients. Whenever multiple responses were received from a facility, responses were used based on position hierarchy.

## Results

**Figure 1.**
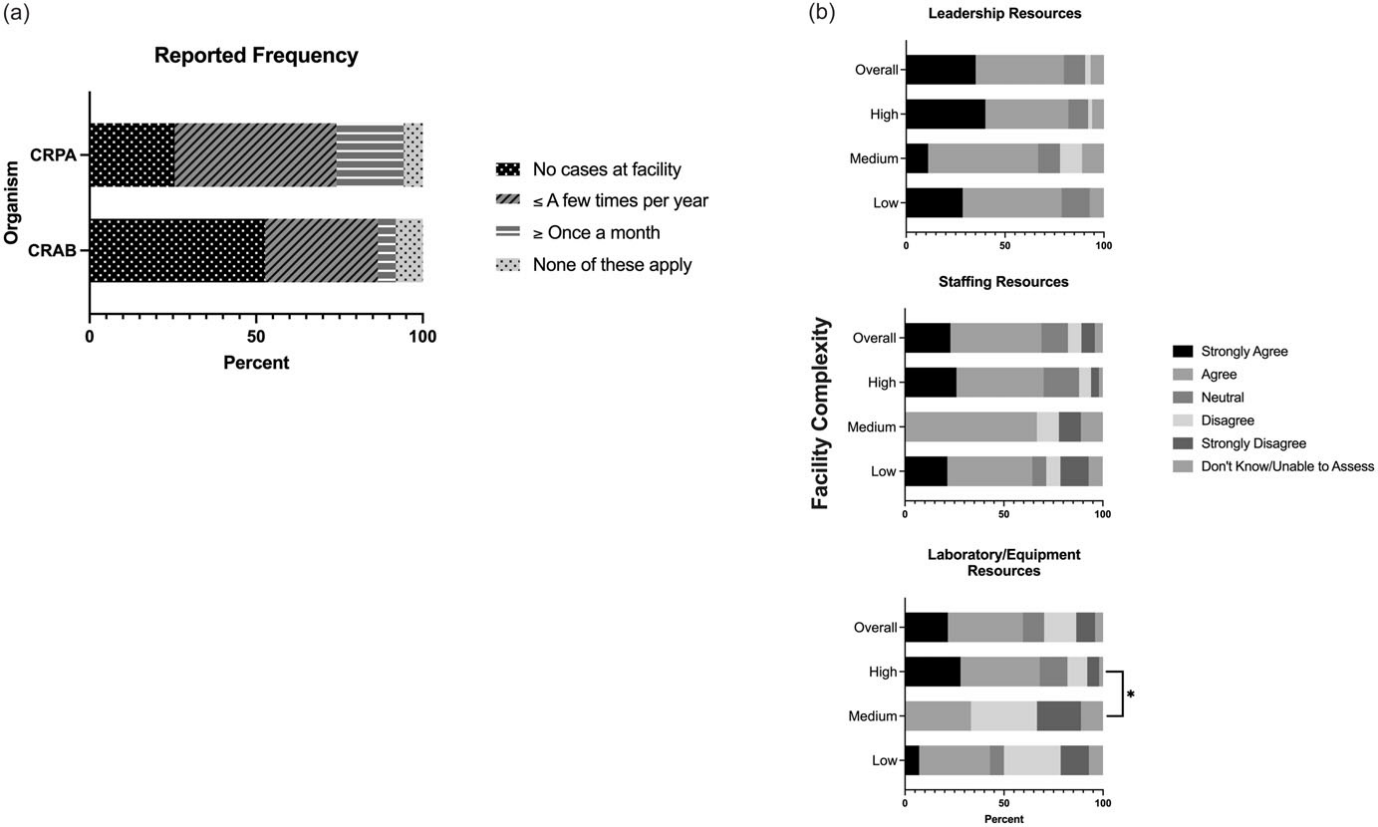
Reported carbapenem-resistant *Pseudomonas aeruginosa* (CRPA) and *Acinetobacter baumannii* (CRAB) incidence and resources available for testing by facility complexity and location. n = 74 unique VAMC responses. (***a***) Reported CRPA and CRAB incidence. No significant differences in incidence between facility complexities. (***b***) Reported leadership, staffing, and laboratory and equipment resources available for CRAB and CRPA testing. Significant differences in reported laboratory/equipment resources between high and medium-complexity facilities (68% vs 33.3%, *P* = 0.027).

89 survey responses across 74 unique facilities (58.7% response rate) were received from mostly lead or supervisory laboratory technologists (23.3% and 60.3%, respectively). Most responses were from urban (90.6%) and high-complexity facilities (67.6%), with no significant difference in complexity (*P* = 0.49) or location (*P* = 0.27) found between responders and nonresponders. 53.3% of facilities reported no CRAB detection and ∼one-third reported seeing CRAB ≤ few times per year (Figure 1a). Only 5% of all responses reported detecting CRAB ≥ once per month. A greater proportion of low-complexity facilities (73.3%) reported no CRAB cases compared to high-complexity facilities (46%), although this was not statistically significant. CRPA was reported more frequently, with only 25.6% of facilities reporting no cases compared to 48.6% reporting ≤ few times per year and 20.3% reporting ≥ once per month.

Nearly 90% of respondents reported the use of a recommended method such as minimum inhibitory concentration and disk diffusion tests with carbapenem for primary identification with 10% of labs using off-site testing services (Table 1). Around 20% of respondents do not test for carbapenemases when initial testing suggests CRAB or CRPA isolates. Of facilities that do test for carbapenemases, significantly more high-complexity facilities utilize commercial molecular platforms (37.5% of high-complexity vs 4.1% of medium-complexity and 2.7% of low-complexity facilities, *P* = 0.02). 30.7% of labs utilize reference labs or other VAMCs. Testing methods were not significantly different between urban and rural facilities. One-third of labs report performing on-site testing, most of which are high-complexity facilities (42.0% of high-complexity vs 11.1% of medium-complexity vs 13.3% of low-complexity facilities, *P* = 0.05). Commercial and public reference labs are also used (14.7% and 29.3%, respectively). 24% of labs perform additional antibiotic susceptibility testing following the detection of CRAB and CRPA, with most testing for susceptibility to combination antibiotics. No significant differences in testing methods or additional antibiotic susceptibility testing were identified between facility complexity or rurality.

**Table 1.** Summary of survey responses and statistics by facility complexity and location

		Facility complexity				Facility location		
Survey responses	Number of responses	High (n=50)	Medium (n=9)	Low (n=15)	***P*** **value**	Rural (n=7)	Urban (n=67)	***P*** **value**
**Carbapenem-resistant** ***Acinetobacter baumannii***								
No cases at facility	39	23 (46.0%)	5 (55.6%)	11 (73.3%)	0.33	6 (85.7%)	33 (49.3%)	0.65
≤ A few times per year	25	21 (42.0%)	3 (33.3%)	1 (6.7%)		1 (14.3%)	24 (35.8%)	
≥ Once a month	4	3 (6.0%)	0 (0.0%)	1 (6.7%)		0 (0.0%)	4 (6.0%)	
None of these apply	6	3 (6.0%)	1 (11.1%)	2 (13.3%)		0 (0.0%)	6 (9.0%)	
**Carbapenem-resistant** ***Pseudomonas aeruginosa***								
No cases at facility	19	12 (24.0%)	1 (11.1%)	6 (40.0%)	0.27	3 (42.9%)	16 (23.9%)	0.46
≤ A few times per year	36	25 (50.0%)	4 (44.4%)	6 (40.0%)		4 (57.1%)	31 (46.3%)	
≥ Once a month	15	11 (22.0%)	3 (33.3%)	1 (6.7%)		0 (0.0%)	15 (22.4%)	
None of these apply	5	2 (4.0%)	1 (11.1%)	2 (13.3%)		0 (0.0%)	5 (7.5%)	
**Laboratory practices**								
Use MIC or disk diffusion for primary identification	66	48 (96.0%)	8 (88.9%)	10 (66.7%)	0.31	5 (71.4%)	61 (91.0%)	0.61
Perform confirmatory testing	56	37 (74.0%)	7 (77.7%)	12 (80.0%)	0.76	5 (71.4%)	51 (76.1%)	0.66
Confirm carbapenemase production	57	39 (78.0%)	8 (88.9%)	10 (66.7%)	0.9	4 (57.1%)	53 (79.1%)	0.62
Use commercial molecular platform for carbapenemase production testing	32	27 (54.0%)	3 (33.3%)	2 (13.3%)	**0.01**	30 (44.8%)	2 (28.6%)	0.69
Perform additional antibiotic susceptibility testing^a^	18	15 (30.0%)	3 (33.3%)	0 (0.0%)	0.22	1 (16.7%)	16 (24.2%)	0.85
Perform on-site testing	25	21 (42.0%)	1 (11.1%)	2 (13.3%)	**0.04**	1 (14.3%)	23 (34.3%)	0.42
**Resource availability (“Strongly Agree” and “Agree” responses)**								
Leadership	58	41 (82.0%)	6 (66.7%)	11 (88.6%)	0.55	5 (71.4%)	53 (80.3%)	0.16
Staffing	50	35 (70.0%)	6 (66.7%)	9 (64.3%)	0.24	4 (57.1%)	46 (69.7%)	0.06
Laboratory/equipment	43	34 (68.0%)	3 (33.3%)	6 (42.9%)	**0.03**^**b**^	3 (42.9%)	40 (60.6%)	0.26

Note. MIC, minimum inhibitory concentration.

a Tobramycin, amikacin, polymyxin B, colistin, tigecycline, ceftazidime-avibactam, ceftolozane-tazobactam, meropenem-vaborbactam, imipenem-relebactam, aztreonam-avibactam, cefiderocol.

b Significant difference between high- and medium-complexity facilities.

Resources available for CRAB and CRPA testing were also surveyed (Figure 1b). More than 75% of facilities agreed that local leadership provided the resources necessary for CRAB and CRPA testing, and 67.5% of facilities overall agreed that their facility had the staffing resources necessary. 58% of facilities agreed they had sufficient laboratory and equipment resources for testing, with a significantly greater proportion of high-complexity facilities reporting sufficient resources compared to medium-complexity facilities (68.0% vs 33.3%, *P* = 0.03) and low-complexity facilities in analyses where low and medium complexities were combined (no other additional significant differences identified). No difference in leadership or staffing resources by complexity or location and no difference in laboratory resources was identified between rural and urban facilities.

## Discussion

Laboratory knowledge of testing procedures and consistent protocols are essential for the rapid identification of MDRO pathogens and implementation of infection control measures. We found that the reported incidence of CRAB is low, though may be clinically significant, while CRPA cases were reported more frequently.

Most laboratories surveyed are performing similar, recommended tests for primary identification and carbapenemase production testing.^9^ Most high-complexity facilities utilize commercial molecular platforms to perform carbapenemase testing, with significantly fewer medium- and low-complexity facilities reporting use. This may be tied to the availability and prioritization of laboratory resources at lower complexity facilities, particularly given the current guidelines that suggest routine testing for resistance genes in CRAB are not recommended.^6^ Significantly more high-complexity facilities perform on-site testing compared to medium and low. With the availability of state and national reference labs enabling more widespread MDRO testing, the ability to perform on-site testing may not be required for VAMCs.^10^

Availability of leadership support, laboratory, and staffing resources influences MDRO testing abilities. Most VAMC laboratories report sufficient leadership and staffing resources for CRAB and CRPA testing at their facilities. However, medium-complexity facilities reported significantly lower availability of laboratory and equipment resources. Medium-complexity facilities may feel at higher risk for these organisms and/or more compelled than low-complexity facilities to test for these organisms in-house. Note, however, that the difference in perceived resources between low- and high-complexity facilities may have been statistically nonsignificant due to small samples. Nevertheless, medium-complexity facilities may benefit from being directed to external reference laboratories.

Though a national sample was surveyed, results may not be generalizable to non-VA hospitals. Our moderate sample size may not have provided sufficient power to detect differences in responses. Most responses to the survey were from high-complexity facilities in urban locations, which may limit our conclusions in rural locations and facilities with medium- and low-complexity patients. Additionally, recall bias in responses may influence the accuracy of results.

Ultimately, most VAMCs surveyed do not currently report a high incidence of CRAB and CRPA and use similar testing and identification protocols. A large portion of VAMCs utilize off-site testing resources, likely enabling more widespread testing. These results indicate that there is currently time to develop and optimize recommendations for VA laboratories to improve future testing for CRAB and CRPA. Implementation of consistent and equitable testing and identification protocols now, when reported incidence is relatively low, will be essential to prevent widespread transmission of CRAB and CRPA in VAMCs.

## Supporting information

Sawyer et al. supplementary material Sawyer et al. supplementary material

## Supplementary material

For supplementary material accompanying this paper visit http://doi.org/10.1017/ash.2024.404. click here to view supplementary material
