# Supplementary material for: A cross-sectional survey of Department of Veterans Affairs laboratory practices for identification of carbapenem-resistant Acinetobacter baumannii and Pseudomonas aeruginosa
Source: Antimicrob Steward Healthc Epidemiol. 2024 Nov 11;4(1):e197. doi: 10.1017/ash.2024.404 (PMC11574601; doi:10.1017/ash.2024.404)
Supplement: Sawyer et al. supplementary material [file S2732494X24004042sup001.pdf]

SURVEY QUESTIONS FOR LABORATORIANS

VA QUERI CARRIAGE II Program Survey of laboratory practices of Carbapenemase-producing and Carbapenem-resistant Acinetobacter baumannii and Pseudomonas aeruginosa

This survey is focused on understanding laboratory practices related to carbapenemase-producing and carbapenem-resistant Acinetobacter baumannii and Pseudomonas aeruginosa in your facility. You are being asked to participate because of your role in laboratory surveillance. We encourage you to seek input as needed from other staff for questions you are unfamiliar or unable to answer. This survey is voluntary, and responses will be kept confidential. Identifiers will not be shared outside the CARRIAGE II team. The survey should take 5-10 minutes to complete.

---

1. VISN number:

◯ 1
◯ 2
◯ 4
◯ 5
◯ 6
◯ 7
◯ 8
◯ 9
◯ 10
◯ 12
◯ 15
◯ 16
◯ 17
◯ 19
◯ 20
◯ 21
◯ 22
◯ 23

2. Facility and station number (If you are not sure what your station number is, you may look it up here: https://www.va.gov/directory/guide/rpt_fac_list.cfm):

◯ 402
◯ 405
◯ 436
◯ 436A4
◯ 437
◯ 438
◯ 442
◯ 459
◯ 460
◯ 463
◯ 501
◯ 502
◯ 503
◯ 504
◯ 506
◯ 508
◯ 509
◯ 509A0
◯ 512
◯ 512A5
◯ 512GD
◯ 515
◯ 516
◯ 517
◯ 518
◯ 519
◯ 520
◯ 521
◯ 523
◯ 523A4
◯ 523A5
◯ 526
◯ 528
◯ 528A4
◯ 528A5
◯ 528A6
◯ 528A7
◯ 528A8
◯ 529
◯ 529A4
◯ 531
◯ 534
◯ 537
◯ 538
◯ 539
◯ 540
◯ 541
◯ 542
◯ 544
◯ 546
◯ 548
◯ 549
◯ 549A4
◯ 549A5
◯ 550
◯ 552
◯ 553
◯ 554
◯ 554A4
◯ 556
◯ 557
◯ 558
◯ 561
◯ 561A4
◯ 562
◯ 564
◯ 565
◯ 568
◯ 568A4

- ◯ 570
- ◯ 573
- ◯ 573A4
- ◯ 575
- ◯ 578
- ◯ 580
- ◯ 581
- ◯ 583
- ◯ 585
- ◯ 586
- ◯ 589
- ◯ 589A4
- ◯ 589A5
- ◯ 589A6
- ◯ 589A7
- ◯ 590
- ◯ 593
- ◯ 595
- ◯ 596
- ◯ 596A4
- ◯ 598
- ◯ 598A0
- ◯ 600
- ◯ 603
- ◯ 605
- ◯ 607
- ◯ 608
- ◯ 610
- ◯ 610A4
- ◯ 612
- ◯ 612A4
- ◯ 612GF
- ◯ 613
- ◯ 614
- ◯ 618
- ◯ 619
- ◯ 619A4
- ◯ 620
- ◯ 620A4
- ◯ 621
- ◯ 623
- ◯ 626
- ◯ 626A4
- ◯ 629
- ◯ 630
- ◯ 630A4
- ◯ 630A5
- ◯ 631
- ◯ 632
- ◯ 635
- ◯ 636
- ◯ 636A4
- ◯ 636A6
- ◯ 636A8
- ◯ 637
- ◯ 640
- ◯ 640A0
- ◯ 640A4
- ◯ 642
- ◯ 644
- ◯ 646
- ◯ 646A4
- ◯ 648
- ◯ 648A4
- ◯ 649
- ◯ 650
- ◯ 652
- ◯ 653
- ◯ 654
- ◯ 655
- ◯ 656

◯ 657
◯ 657A0
◯ 657A4
◯ 657A5
◯ 658
◯ 659
◯ 660
◯ 662
◯ 663
◯ 663A4
◯ 664
◯ 666
◯ 667
◯ 668
◯ 671
◯ 671A4
◯ 672
◯ 673
◯ 674
◯ 674A4
◯ 675
◯ 676
◯ 678
◯ 679
◯ 687
◯ 688
◯ 689
◯ 691
◯ 691A4
◯ 692
◯ 693
◯ 695

3. How often do you see the following organisms (unique isolates) in your laboratory?

	Once per year	A few times per year	Once a month	Greater than once a month	We have not seen this organism at my VAMC	None of these options apply to my VAMC
Carbapenem-resistant Acinetobacter baumannii	◯	◯	◯	◯	◯	◯
Confirmed carbapenemase-producing Acinetobacter baumannii	◯	◯	◯	◯	◯	◯
Carbapenem-resistant Pseudomonas aeruginosa	◯	◯	◯	◯	◯	◯
Confirmed carbapenemase-producing Pseudomonas aeruginosa	◯	◯	◯	◯	◯	◯

4. What antimicrobial susceptibility testing methods does your lab use as a primary first step to identify a Carbapenem-resistant A. baumannii or P. aeruginosa? Please select the primary test used for each organism. (check all that apply)

	A. baumannii	P. aeruginosa
E-test	☐	☐
Automated MIC/Broth	☐	☐
Kirby Bauer Test	☐	☐
Disk Diffusion	☐	☐
Manual Broth microdilution	☐	☐
Don't know	☐	☐
Testing is done off-site	☐	☐
Other	☐	☐

Please specify (A. baumannii): ______________________________

Please specify (P. aeruginosa): ______________________________

5. If your primary step suggests a Carbapenem-resistant Acinetobacter baumannii or Pseudomonas aeruginosa, what method does your laboratory use to confirm the initial susceptibility testing? (check all that apply)

	A. baumannii	P. aeruginosa
Repeat first test	☐	☐
Automated MIC/Broth	☐	☐
Kirby Bauer Test	☐	☐
Disk Diffusion	☐	☐
Manual Broth microdilution	☐	☐
Don't know	☐	☐
We don't do a second round of susceptibility testing for this organism(s)	☐	☐
Other	☐	☐

Please specify (A. baumannii): ______________________________

Please specify (P. aeruginosa): ______________________________

6. If the results of carbapenem susceptibility testing for A. baumannii or P. aeruginosa suggest a possible carbapenemase-producing Acinetobacter baumannii or carbapenemase-producing Pseudomonas aeruginosa, which method(s) does your lab or your reference lab use to confirm that the Acinetobacter baumannii or Pseudomonas aeruginosa isolate produces a carbapenemase? (check all that apply)

	A. baumannii	P. aeruginosa
Modified Hodge Test (MHT)	☐	☐
Modified carbapenem inactivation method (mCIM)	☐	☐
CarbaNP	☐	☐
Carba-R	☐	☐
Other PCR detection from the initial culture (please specify which genes are detected between a-f)	☐	☐
a. KPC	☐	☐
b. NDM	☐	☐
c. IMP	☐	☐
d. VIM	☐	☐
e. OXA-48	☐	☐
f. Other OXA genes	☐	☐
Performed in reference lab or another VA and unsure of method used	☐	☐
Don't know	☐	☐
We don't perform or send carbapenemase testing for this organism(s)	☐	☐
Other	☐	☐

Please specify here (A. baumannii): ______________________

Please specify here (P. aeruginosa): ______________________

6a. Where is the confirmatory testing performed? (Check all that apply)

	A. baumannii	P. aeruginosa
On site/In house	☐	☐
Commercial Reference Laboratory (e.g., Quest, LabCorp, etc)	☐	☐

Public Reference Laboratory (e.g., public health department, CDC Antimicrobial Resistance Laboratory Network lab)	☐	☐
University Affiliate Laboratory	☐	☐
Another VA Medical Center lab	☐	☐
Don't know	☐	☐
Not applicable-No confirmatory testing is done.	☐	☐

7. If your lab tests for carbapenemase production for Acinetobacter baumannii and/or Pseudomonas aeruginosa, do you amend the carbapenem susceptibility interpretations in any way if a carbapenemase is detected?

	Yes	No	Don't know	Not Applicable
A. baumannii	◯	◯	◯	◯
P. aeruginosa	◯	◯	◯	◯

8. If your lab tests for carbapenemase production for Acinetobacter baumannii and/or Pseudomonas aeruginosa, do you amend the beta-lactam susceptibility interpretations in any way if a carbapenemase is detected?

	Yes	No	Don't know	Not Applicable
A. baumannii	◯	◯	◯	◯
P. aeruginosa	◯	◯	◯	◯

9. If your lab identifies a carbapenemase-producing Acinetobacter baumannii or carbapenemase-producing
Pseudomonas aeruginosa, does your lab (or reference lab) perform antibiotic susceptibility testing on any
additional agents per lab protocol?

	Yes	No	Don't know	Not Applicable
A. baumannii	◯	◯	◯	◯
P. aeruginosa	◯	◯	◯	◯

9a. If your lab (or reference lab) performs additional antibiotic susceptibility testing for carbapenemase-producing Acinetobacter baumannii or carbapenemase-producing Pseudomonas aeruginosa, which of the
following antibiotics does your lab subsequently report per lab protocol? (check all that apply)

	A. baumannii	P. aeruginosa
Tobramycin	☐	☐
Amikacin	☐	☐
Polymyxin B	☐	☐

Colistin	☐	☐
Tigecycline	☐	☐
Ceftazidime-avibactam	☐	☐
Ceftolozane-tazobactam	☐	☐
Meropenem-vaborbactam	☐	☐
Imipenem-relebactam	☐	☐
Aztreonam-avibactam	☐	☐
Cefiderocol	☐	☐
N/A	☐	☐
Other	☐	☐

Please specify here (A. baumannii): ______________________

Please specify here (P. aeruginosa): ______________________

10. Please note the extent to which you agree or disagree with the following statements related to the detection and confirmation of carbapenemase-producing Acinetobacter baumannii or carbapenemase-producing Pseudomonas aeruginosa:

	Strongly Agree	Agree	Neutral	Disagree	Strongly Disagree	Don't know/ Unable to Assess
a. The local leadership at my facility provides, or would provide (if available), the resources that are needed to support detection and confirmation of these organisms in house or as send-out testing	○	○	○	○	○	○
b. We have the staffing resources we need to accomplish the activities related to detection and confirmation of these organisms in house or as send-out testing	○	○	○	○	○	○
c. We have the laboratory/equipment resources to accomplish the activities related to detection and confirmation of these organisms in house	○	○	○	○	○	○

11. What is your professional title/position? (check all that apply)	☐ Laboratory Technologist (non-Supervisory)<br>☐ Lead Laboratory Technologist<br>☐ Supervisory Laboratory Technologist<br>☐ Microbiology Laboratory Director<br>☐ CLIA Laboratory Director<br>☐ Other
Please specify:	__________________________________
12. How long have you been at this facility? (choose one)	◯ < 1 year<br>◯ 1-5 years<br>◯ 6-10 years<br>◯ 11-15 years<br>◯ 16-20 years<br>◯ 21+ years
